# Regulatory T cells in bullous pemphigoid: biological characteristics, dysfunction, and pathogenic roles

**DOI:** 10.3389/fimmu.2026.1775932

**Published:** 2026-04-21

**Authors:** Zhimin Wang, Yu Lu, Huan Cui, Yiwen Zhang, Lingzhi Meng, Wenyu Chang, Xuesong Yang, Jianzhou Ye

**Affiliations:** 1 First School of Clinic Medicine, Yunnan University of Traditional Chinese Medicine, Kunming, Yunnan, China; 2 Department of Dermatology, Kunming Municipal Hospital of Traditional Chinese Medicine, Kunming, Yunnan, China; 3 Department of Dermatology, First Affiliated Hospital of Yunnan Traditional Chinese Medicine University, Kunming, Yunnan, China

**Keywords:** bullous pemphigoid, Foxp3, immune tolerance, immunology, regulatory T cells

## Abstract

Bullous pemphigoid (BP) is an autoimmune blistering disorder that predominantly affects the elderly. Its pathogenesis involves the disruption of the basement membrane zone (BMZ), driven by pathogenic autoantibodies and a pronounced inflammatory response. Current first-line therapies, primarily based on glucocorticoids and immunosuppressants, are limited by substantial side effects and frequent disease recurrence. Regulatory T cells (Tregs) play a central role in maintaining immune homeostasis and peripheral tolerance, exerting control over effector immune cells through transcriptional regulation, surface markers, and diverse immunosuppressive mechanisms. Accumulating evidence indicates that numerical reduction and functional impairment of Tregs are critical to the breakdown of immune tolerance in BP. Treg dysfunction leads to the aberrant activation of Th2, Th17, and follicular helper T (Tfh) cells, which in turn promotes the production of anti−BP180/BP230 autoantibodies and disrupts the regulation of inflammatory cells such as neutrophils and eosinophils. These events collectively result in BMZ degradation and blister formation. Multiple factors, including age−related immunosenescence, genetic predisposition, pharmacological exposures, and environmental stimuli, can further compromise Treg function, thereby contributing to BP pathogenesis. A deeper understanding of Treg−driven mechanisms in BP provides a rational basis for developing targeted therapeutic strategies.

## 1 Introduction

Bullous pemphigoid (BP) is the most prevalent autoimmune blistering disorder, primarily affecting the elderly population. Its global annual incidence has shown a progressive upward trend. The typical clinical manifestations of BP include widespread tense blisters and erythema across the body, often accompanied by intense pruritus. The underlying pathology is characterized by an abnormal immune response of the immune system to components of the dermal-epidermal BMZ, primarily BP180 and BP230. Approximately 90% of patients exhibit specific autoantibodies targeting the NC16A extracellular non-collagenous domain of BP180 in their serum (1). As the direct source of these pathogenic autoantibodies, B cells play a central role in BP pathogenesis (2). Studies have shown that antibody-secreting cells (plasma cells) are significantly increased in both the circulation and skin lesions of BP patients, and this increase positively correlates with disease severity, directly confirming that B cell activation and autoantibody production are key drivers of the disease (3). These autoantibodies deposit at the dermal-epidermal junction, triggering a complex cascade of events that includes complement activation, inflammatory cell recruitment, and protease release. This cascade ultimately disrupts the structure of the BMZ, leading to subepidermal blister formation (4), while simultaneously causing an imbalance in the local inflammatory microenvironment. Currently, the first-line treatment for BP primarily involves the systemic administration of glucocorticoids in combination with immunosuppressants (5, 6). However, the long-term use of glucocorticoids is associated with severe side effects such as infections, metabolic disorders, and osteoporosis, as well as a high recurrence rate. This highlights the significant contradiction between existing therapies in achieving disease eradication and ensuring long-term safety.

Tregs serve as essential regulators of immune homeostasis and peripheral tolerance, precisely modulating the activation and function of effector immune cells through various mechanisms. They play an irreplaceable role in preventing autoimmune responses. Recent studies have demonstrated that a decrease in Treg quantity, functional defects, and phenotypic instability are critical factors contributing to the breakdown of immune tolerance in BP (**Table 1**). These abnormalities directly facilitate the production of autoantibodies and exacerbate inflammatory damage. However, the reasons behind the failure of Treg mechanisms in maintaining skin immune tolerance under physiological conditions in BP, as well as how the disease microenvironment drives Treg dysfunction, remain inadequately explained. This article aims to comprehensively review the existing evidence, beginning with the biological characteristics of Tregs, to analyze their functional defects in BP, the causal relationship with disease progression, and the internal and external factors influencing their function. The goal is to provide new theoretical perspectives and research approaches for a deeper understanding of BP pathogenesis.

**Table 1:** 

No	Author	Year	Sample	Detection method	Maker	Peripheral circulation	Local skin lesions	Treg function
1	Quaglino P et al. (58)	2012	BP patients (n=26); Healthy controls (n=30)	Flow cytometry	nTreg(CD4+ CD25^bright^ FOXP3+ Treg) aTreg(treg(CD4+ CD25^bright^ FOXP3^-^) CD8+ Treg	nTreg↓ Activated Treg ↑↑	–	The reduced expression of CCR4 and CD62L by CD4+ nTreg cells is associated with skin homing and strong suppressive activity.
2	Hanafusa T et al. (59)	2012	A case of lichen planus-type chronic graft-versus-host disease (cGVHD) complicated with mucous membrane pemphigoid and positive for anti-BP180/BP230 antibodies (n=1)	Flow cytometry	CD4+CD25+Foxp3+ Tregs Foxp3^low^、CD45RA+nativeTregs	↓	–	
3	Antiga E et al. (60)	2014	BP(n=10) HC(n=30)	Flow cytometry Immunohistochemical staining	CD4+CD25+ Tregs CD4+CD25^bright^FOXP3+	↓↓, Elevation after hormone therapy	↓↓,Elevation after hormone therapy	Cannot effectively inhibit the activation and proliferation of self-reactive T cells
4	Antiga E et al. (55)	2014	Dermatitis herpetiformis(n=10) BP (n=10) HC(n=6)	Flow cytometry Immunohistochemical staining	CD4+,CD25+, FOXP3+,TGF-β+, IL-10+; CD4+CD25^bright^FOXP3+	CD4+CD25^bright^FOXP3+Treg ↓↓	FOXP3+/CD4+cell ↓↓ IL-10+/CD4+ cell ↓↓	
5	Marques-Piubelli ML (61)	2023	Immune-related adverse events of bullous pemphigoid (BP-irAE) in cancer patients treated with ICI (n=8) Primary BP (n=8)	Immunohistochemical staining	FOXP3	–	a markedly lower Treg density at the blister floor in the BP-irAE samples when compared with *de novo* BP control samples	
6	Xue R et al. (62)	2024	PBMC from BP patients in the IL-2 treatment group (n=13) and control group (n=8)	Flow cytometry	CD25+ FOXP3+	↓↓,A significant increase in Treg cells was observed after IL-2 treatment.		
7	Terras S et al. (63)	2014	Tissue samples collected from patients with psoriasis (n = 11), vitiligo (n = 10), pemphigus vulgaris (n = 6), BP (n = 8), halo nevi (n = 7), and HC (n = 16)	Immunohistochemical staining	FOXP3		FOXP3+ Treg↑↑ FOXP3+/CD3+T cell↑	
8	Gambichler T et al. (64)	2017	BP (n=31) HC(n=28)	Flow cytometry Immunohistochemical staining	Tregs(CD4+CD25++CD127- Tregs) nTregs(CD4+CD25^++^CD127- FOXP3+) CD4, FOXP3	The percentage of CD4+CD25++CD127- Tregs↑↑	FOXP3+cell ↑↑	
9	Muramatsu K et al. (65)	2020	cBP (before treatment): (n=18) cBP (after treatment) (n = 8):. Elderly healthy controls (n = 12). Young healthy controls (n = 19	Flow cytometry	CD4+Foxp3+T cell aTreg(CD45RA-Foxp3^hi^) nTreg(CD45RA+Foxp3^lo^) CD45RA-Foxp3^lo^ Tcell	The frequencies of untreated cBPCD4+Foxp3+Treg), effector Treg, naive Treg, and CD45RA-Foxp3lo T cells are significantly increased (↑↑), and decrease after systemic corticosteroid treatment	↑↑, After steroid treatment, the frequency of total Treg cells decreases significantly as the disease severity reduces.	Effector Treg is positively correlated with BPDAI, showing compensatory expansion but insufficient function.
10	Rensing-Ehl A et al. (66)	2023	BP(n=13) HC(n=14)	Flow cytometry	CD4+ CD25^low^ CD4+ CD25^high^ CD4+ CD25+FOXP3+Tregs	There is no significant difference between the proportions and absolute quantities.	Infiltration of CD4+, CD25+, and FOXP3+ cells	CD4+CD25 ^high^ Treg cells in peripheral blood express CLA
11	Zhang Y et al. (67)	2024	A patient with bullous pemphigoid (BP) induced by PD-1/PD-L1 inhibitors (n=1)	Multicolor flow cytometry	CD4+CD25+FOXP3+ CD4+ CD25^bright^FOXP3+	↑↑,The absolute count of regulatory T cells decreases after low-dose IL-2 treatment	–	–
12	Hu Z et al. (68)	2024	Skin samples: HC (n=3), PV (n=3), BP (n=4); Peripheral blood samples: HC (n=4), BP (n=3), PV (n=3)	scRNA-seq Flow cytometry	Resting Treg cells (Foxp3low CD45RA+). Activated Treg cells (Foxp3high CD45RA-). Non-suppressive T cells (Foxp3^low^ CD45RA−)	Treg cells, along with CD4+ Treg, CD8+ Tem, and CD8+ Tm, are among the major T cell subsets that exhibit a large number of unique TCR clonotypes.	CD4+ Treg↑↑,	High expression of CTLA4, TIGIT, TOX, PD-1, and functional exhaustion
13	Muramatsu K et al. (69)	2018	Scurfy mice with Foxp3 gene mutation (n=10, n=5) IPEX syndrome patients(n=5) BP (n=1)	Genotype identification	–	Defects in Treg cell function lead to the spontaneous production of IgG autoantibodies against BP230 and type XVII collagen (COL17), and adoptive transfer of CD4+ T cells can induce the production of autoantibodies against full-length COL17 and BP230-1.		
14	Haeberle S et al. (70)	2018	B6.Cg-Foxp3^sf/Y^ (scurfy) mice; WT mice; B6.Cg-Foxn1^nu/J^ (B6/nude) mice; B6.129S7-Rag1^tm1Mom/J^ (B6/RAG) mice.	Genotype identification	Foxp3	The deficiency of Treg cells leads to the production of pathogenic autoantibodies against BP230 (bullous pemphigoid antigen 230), thereby causing subepidermal autoimmune blistering diseases.		

## 2 Biological characteristics of Treg cells

### 2.1 Development and transcriptional regulation of Treg cells

Treg cells play a crucial role in maintaining immune tolerance. Research on immune tolerance can be traced back to the pioneering work of scholars such as Owens, Burnet, and Medawar in the 1950s (7). This concept was first observed by Owen in dizygotic twin cattle, where placental vascular anastomosis led to hematopoietic chimerism, resulting in the absence of antibody responses upon mutual blood transfusion—in contrast to the clear antibody responses observed in normal adult cattle (8). Subsequently, Medawar’s team further demonstrated the inducibility of immune tolerance through chicken parabiosis experiments and neonatal murine allograft transplantation, proposing that immune tolerance is an acquired state “learned” by the immune system through antigen exposure during development (9, 10). Based on these findings, Burnet proposed the clonal selection theory, providing a core theoretical framework for immune tolerance: lymphocytes that encounter antigens during development undergo clonal deletion, thereby establishing tolerance to self-antigens (11). Subsequent studies have confirmed that the adoptive transfer of Treg cells can induce long-term tolerance to grafts and other entities, with this effect being maintained and enhanced through the recruitment and differentiation of additional inhibitory cells (12–14). The critical role of Tregs in immune homeostasis is underscored by the fact that their absence triggers widespread autoimmune diseases in mice, while their reinfusion can delay disease progression (15, 16). The development of Treg cells begins during the embryonic stage and primarily occurs in the thymus. Tregs can be classified into thymus-derived natural Tregs (tTregs), peripherally induced Tregs (pTregs), and *in vitro*-induced Tregs (iTregs) based on their origin (17, 18). The development of tTregs depends on T cell receptors (TCRs) recognizing self-antigen peptide-MHC class II molecule complexes on thymic stromal cells with relatively high affinity (19). During this process, cells that receive sustained strong TCR signals tend to differentiate into autoreactive effector T cells, whereas those with relatively weak TCR signals or regulated by factors such as TGF-β initiate Forkhead box protein P3(Foxp3) expression and become committed to the Treg lineage under the influence of local IL-2 (20, 21). Mature tTregs can be further classified based on their activation status into central regulatory T cells (cTreg, also known as resting Tregs or rTreg) (22, 23) in the initial quiescent state, effector regulatory T cells (eTreg, also known as activated Tregs or aTreg) in the activated state, and memory regulatory T cells (mTreg) that efficiently exert inhibitory functions upon re-exposure to the same antigen (22, 24, 25). pTregs are primarily generated from naive CD4+ T cells in response to cytokines such as TGF-β and IL-10, particularly within the intestinal mucosa. Their phenotype is generally less stable than that of tTregs (26–28). Studies indicate that tTregs exhibit superior functional persistence compared to their peripheral counterparts, making them a more ideal source for therapeutic applications (29).

Foxp3 serves as the core transcription factor for Treg development, phenotypic maintenance, and functional execution, sustaining suppressive activity by regulating the expression of key molecules such as CTLA-4, CD25, and GITR (30). It possesses both transcriptional activation and repression capabilities, promoting CD25 expression while inhibiting IL-2 transcription, which contributes to the characteristic dependence of Tregs on exogenous IL-2 (31). The absence or dysfunction of Foxp3 expression results in a complete loss of immunosuppressive capacity in Treg cells. Notably, Scurfy mice with Foxp3 gene mutations and human patients with immune dysregulation, polyendocrinopathy, enteropathy, X-linked (IPEX) syndrome (32) exemplify this loss, as they develop multi-organ inflammation, including autoimmune skin damage, due to the lack of functional Treg cells. This directly demonstrates the indispensable role of Foxp3 in maintaining immune tolerance. Furthermore, transcription factors such as NFAT and STAT5 are involved in regulating the expression of Treg-related genes, influencing their functional stability (33). Treg cells may exhibit varying transcription factor expression profiles under different microenvironmental conditions, thereby affecting their functional outcomes and fate determination (34). The interactions and regulatory mechanisms of these transcription factors not only provide crucial insights into the biological characteristics of Treg cells but may also present novel therapeutic targets for related diseases.

### 2.2 Characteristic surface markers of Treg cells

The immunosuppressive function of Treg cells is intricately linked to their specific surface markers. These molecules not only act as identity markers but also play a crucial role in regulating their development, stability, and functional execution. Key surface markers include FoxP3, CD4, CD25, CTLA-4, and PD-1, among others. CD4 functions as a co-receptor for T cells, thereby classifying them as a subset of helper T cells. The elevated expression of CD25 (IL-2 receptor α chain) enables Treg cells to efficiently compete for and uptake IL-2 within the microenvironment, which is vital for maintaining their survival and expansion while simultaneously limiting IL-2 availability for effector T cells. FoxP3, as the lineage-determining transcription factor, is essential for sustaining the suppressive phenotype and function of Treg cells, with its expression directly regulating a cascade of downstream functional molecules. The absence or mutation of FoxP3 results in the loss of Treg cell function, subsequently triggering autoimmune diseases, thereby underscoring its irreplaceable role (35, 36). In addition to the core molecules that form their identity foundation, a range of inducible molecules further enhances the functional plasticity and environmental adaptability of Tregs. For example, activated Tregs upregulate HLA-DR, and this subset may demonstrate dysfunction in autoimmune diseases such as systemic lupus erythematosus, correlating with disease activity (37). The high expression of tumor necrosis factor receptor 2 (TNFR2) is linked to the potent suppressive function and proliferative advantage of Tregs. The TNFR2+ Treg subset, enriched in the tumor microenvironment, serves as a critical factor in promoting immune evasion, thus emerging as a potential therapeutic target (34, 38). Treg cells express immune checkpoint molecules such as CTLA-4 and PD-1, either constitutively or inducibly, which directly mediate their contact-dependent suppressive functions. Clinical studies have shown that blocking the CTLA-4 or PD-1/PD-L1 pathways can partially attenuate Treg activity, thereby enhancing anti-tumor immune responses (39, 40). Consequently, research on Treg cell surface markers not only establishes a foundation for understanding their biological characteristics but also provides crucial references for clinical applications and the development of therapeutic strategies.

### 2.3 Conservation and differences between human and mouse Tregs

Human and mouse Tregs are highly conserved with respect to their core function of maintaining immune tolerance, yet exhibit important differences in phenotypic markers, molecular regulation, and functional mechanisms. Both subsets utilize the transcription factor FOXP3 as a key regulatory hub and express high levels of CD25 to respond to IL-2 signals essential for their survival and function (41, 42). They also possess contact-dependent suppressive capacity, effectively inhibiting effector T cell proliferation (43, 44) through molecules such as CTLA-4 (45). However, species-specific differences exist in FOXP3 expression regulation: for example, FOXP3 expression in human Tregs can be induced upon activation, and their phenotype exhibits greater heterogeneity (41). Studies have shown that co-expression of CD25 and TNFR2 more effectively identifies functionally potent Treg populations in human peripheral blood, resulting in identification of five times more FOXP3+ cells (46) than with the CD25^high^ standard alone. In mice, only a subset of Foxp3+ T cells possess potent suppressive capacity. TNFR2 mediates tumor necrosis factor (TNF) activation on Tregs and serves as an excellent biomarker for the most inhibitory subgroup of Tregs in mice (41). Glycoprotein A repetitions predominant protein (GARP), which anchors latent transforming growth factor-β1 to the cell surface, is primarily considered a marker of activated Tregs in humans. In contrast, GARP is constitutively expressed at low levels on resting mouse Tregs and can be upregulated by IL-2 or IL-4 alone, independent of T cell receptor signaling (47). Furthermore, GARP deficiency has limited impact on mouse Treg suppressive function in tumor models, contrasting with findings that antibodies targeting GARP on human Tregs effectively inhibit their function (47, 48). The effects of Toll-like receptor 2 activation on human and mouse Tregs also differ: studies indicate that TLR2 agonists enhance human Treg suppressive function. While early studies suggested that TLR2 agonists reverse mouse Treg suppression, subsequent rigorous experiments have confirmed that TLR2 agonists do not reverse mouse Treg suppressive function but instead promote their survival through induction of Bcl-xL (43).

## 3 Immunosuppressive mechanisms of Treg cells

The direct suppressive function of Tregs primarily relies on cell-to-cell contact. Firstly, their highly expressed CTLA-4 binds with high affinity to the co-stimulatory molecules CD80 and CD86 on the surface of antigen-presenting cells (APCs). This interaction not only competitively blocks the CD28-B7 co-stimulatory signal but also actively downregulates CD80 and CD86 on the APC surface through trans-endocytosis, significantly impairing the antigen-presenting capacity of APCs and suppressing the activation of naive T cells at the source (49, 50), Secondly, Tregs can directly interact with effector T cells, particularly CD8+ T cells, by expressing inhibitory receptors such as PD-1 and LAG-3, which inhibit their proliferation and cytotoxicity to prevent excessive immune responses (38, 51). Additionally, the ectoenzymes CD39 and CD73 present on the surface of Tregs catalyze the conversion of pro-inflammatory ATP released in the microenvironment into adenosine. Adenosine acts on the A2A receptors of effector T cells, suppressing their energy metabolism, cell cycle progression, and effector functions, thereby establishing a potent local metabolic inhibitory barrier (50). These direct effects are not limited to classical immune cells. In pathological contexts, such as the tumor microenvironment, the accumulation of Tregs can promote immune escape through similar mechanisms, while their functions are also regulated by secretory factors from tumor cells and cancer-associated fibroblasts, forming a complex regulatory network (37, 52).

In addition to direct contact, Treg cells achieve extensive and enduring immune regulation through the secretion of various inhibitory cytokines. Among these, IL-10, TGF-β, and IL-35 are three pivotal effector molecules (28, 53). IL-10 broadly suppresses the proliferation of effector T cells, the production of pro-inflammatory cytokines, and the functions of antigen-presenting cells. It also regulates complement activation and inhibits the functions of neutrophils and eosinophils (35, 54). In patients with BP, the secretion level of IL-10 is closely associated with disease activity, exhibiting a significantly diminished response to IFN-γ during the active phase (55). TGF-β not only directly inhibits the activation and function of various immune cells but also plays a critical role in inducing the differentiation of naive T cells into peripheral Tregs (pTregs), which is essential for maintaining peripheral tolerance (36). As a recently identified cytokine, IL-35 is specifically produced by Treg cells and effectively suppresses effector T cell responses. Additionally, it induces the generation of inhibitory populations such as regulatory B cells, thereby forming a cascade inhibitory network (37). Treg cells express CD25 at high levels, which competitively consumes IL-2 in the microenvironment with high affinity. This deprives effector T cells of this critical growth factor, essential for their survival and proliferation, thereby indirectly suppressing the expansion of effector immune cells (49, 56, 57). Consequently, a deeper understanding of the interaction mechanisms between Treg cells and other immune cells is crucial for the development of novel immunotherapeutic strategies.

## 4 Evidence for Treg cell deficiencies in BP pathogenesis

### 4.1 Decreased Treg number in peripheral blood and skin lesions of BP patients

Patients with BP exhibit significant abnormalities in the numbers of Tregs, which are distinctly evident in both the circulatory system and lesional tissues. This phenomenon represents a crucial pathological basis for the onset and progression of the disease. A hallmark feature of BP patients is the reduction of circulating Treg cells. Multiple clinical studies have confirmed that active BP patients show significantly lower percentages and absolute counts of CD4+CD25^bright^FOXP3+ Treg cells in peripheral blood compared to healthy individuals (58). Furthermore, the quantity of circulating Treg cells is inversely correlated with disease activity, with more pronounced reductions observed in untreated active-phase patients. Following treatment-induced remission, Treg cell numbers can gradually recover to normal levels (65). Flow cytometry analysis has revealed a significantly increased proportion of circulating CD4+CD25^bright^FOXP3+ activated T cells in BP patients (71), confirming the imbalance in the Treg/effector T cell equilibrium.

Immunohistochemical studies of local lesional tissues have revealed that, although the number of Foxp3+ Treg cells in BP lesions is higher than in healthy skin, it remains significantly lower than in other inflammatory skin diseases such as psoriasis and atopic dermatitis (60). Furthermore, the ratio of Foxp3+ Treg cells to CD4+ T cells is markedly reduced compared to healthy controls (58), indicating a relative insufficiency of local suppressive cells to effectively control inflammation. Single-cell sequencing studies have provided mechanistic insights, demonstrating that Treg cells in BP patients exhibit defective skin migration due to downregulated expression of the chemokine receptor CCR4 on their surface. This downregulation impairs their ability to respond effectively to chemotactic signals, such as CCL22 produced at lesion sites, resulting in inadequate local recruitment (68). Animal experiments have provided direct evidence supporting this mechanism. For example, 12/15-lipoxygenase-deficient mice show significantly reduced Treg recruitment in their skin following the induction of BP-like skin lesions, which leads to persistent aggravation of inflammation (72). More typically, Scurfy mice, which completely lack functional Tregs due to Foxp3 gene mutations, spontaneously develop autoantibodies against BP180/BP230, with some individuals even developing skin blisters (70). This directly confirms the causal relationship between Treg deficiency and BP-like lesions. Although a few early studies failed to identify numerical deficiencies due to differences in detection markers or patient populations (64, 66, 73), these discrepancies may be attributed to variations in detection markers, disease stages, and age, among other factors. However, the majority of large-scale studies in recent years have reached a consensus that reduced Treg numbers constitute one of the core immunopathological features of BP.

### 4.2 Functional impairments of Treg cells in BP

In addition to reduced circulating Treg numbers, BP patients exhibit multi-layered functional defects in Treg cells. Antiga E et al. (60) found that Tregs in BP patients showed decreased FOXP3 expression, impaired CTLA-4 expression, and significantly diminished IL-10 secretion capacity even under anti-CD3/CD28 stimulation (63). Through single-cell sequencing of skin lesions, Zhi Hu et al. (68) further discovered that local Tregs exhibited high expression of exhaustion-related genes including CTLA4, TIGIT, TOX, PDCD1, and LAG3, suggesting that although Treg numbers increase in lesions, their function may be compromised due to chronic antigen stimulation. However, *in vitro* functional assays demonstrated that circulating Tregs from BP patients could still effectively suppress IFN-γ secretion by Tresp upon allogeneic stimulation. This suppressive function was independent of IL-10/TGF-β, consistent with the contact-dependent mechanism of natural Tregs (66). However, this study used alloantigens or unrelated antigens such as tetanus toxoid as stimulation sources, rather than BP autoantigens (BP180/BP230). Therefore, it cannot exclude the possibility of specific Treg functional defects against disease-related autoantigens in patients. Additionally, while the presence of FOXP3+ cell infiltration in lesions was confirmed, the tissue Tregs were not isolated for functional testing. The high local concentration of proinflammatory factors may inactivate infiltrating Tregs or reprogram them into effector phenotypes. Their mere presence in tissues does not equate to normal functionality. Furthermore, dysregulated chemokine receptor expression exacerbates functional impairment through homing defects: reduced expression of CCR4, CCR6 and other receptors on Tregs weakens their ability to migrate to inflamed skin, resulting in insufficient local infiltration in lesions despite normal circulating Treg numbers (74). The most direct evidence comes from the Treg-deficient scurfy mouse model, which spontaneously develops BP-like lesions. Adoptive transfer of healthy mouse Tregs significantly improves symptoms. If BP were solely caused by reduced Treg numbers, exogenous Treg supplementation should have been effective, however, such supplementation cannot fully reverse the disease. This result directly demonstrates the central role of functional impairment in pathogenesis (70, 75).

## 5 Pathological consequences of Treg dysfunction in BP

### 5.1 Defects in Treg function lead to the destruction of immune tolerance, the production of autoantibodies, and the formation of skin lesions

Tregs are the core cell population maintaining peripheral immune tolerance. As the sole source of pathogenic anti-BP180/BP230 autoantibodies, B cells are the central effector cells in BP pathogenesis (3). The abnormal activation and differentiation of B cells into antibody-secreting plasma cells is the key downstream event of immune tolerance breakdown mediated by Treg dysfunction. They effectively suppress the activation of autoreactive CD4+ T cells (particularly Th2 and Tfh cells) by expressing key transcription factors such as Foxp3 and secreting inhibitory cytokines like IL-10 and TGF-β. They also employ CTLA-4-mediated cell contact-dependent suppression mechanisms, thereby maintaining immune tolerance to skin basement membrane antigens such as BP180 (55). In BP, this tolerance mechanism is disrupted in multiple ways. Firstly, characteristic alterations occur in the quantity and functional lineage of Tregs: multiple studies have shown that the percentage and absolute number of CD4+CD25^bright^FOXP3+ Tregs in the peripheral blood of BP patients are significantly lower than in healthy controls (58–60, 67), and this reduction is accompanied by an expansion of the CD4+CD25^bright^FOXP3^−^ activated T cell subset (58). ScRNA-seq further reveals that the increased Tregs in BP skin lesions exhibit high expression of genes associated with T cell exhaustion, such as CTLA4, TIGIT, TOX, PDCD1, and LAG3, suggesting their immunosuppressive function may be impaired (68). Notably, different etiological subtypes of BP may involve distinct patterns of Treg dysfunction: abnormalities in functional effector Tregs (CD45RA^−^Foxp3^hi^) in conventional BP correlate with disease severity, whereas dysregulation of naive Tregs (CD45RA+Foxp3^lo^) is more prominent in dipeptidyl peptidase-4 inhibitor-associated BP, indicating heterogeneity in pathological mechanisms (65). The application of immune-checkpoint inhibitors (ICIs) provides direct evidence for understanding the role of Tregs in BP tolerance breakdown. ICIs enhance anti-tumor immune responses by blocking immune regulatory pathways such as PD-1 and CTLA-4, but simultaneously disrupt Treg-mediated immune tolerance, leading to a reduction in Treg numbers and functional suppression, thereby inducing or exacerbating BP (65, 76). Specifically, after ICI treatment, interactions between Langerhans cells and Tregs in the skin decrease; the local immunosuppressive environment collapses, promoting inflammatory responses and the onset of BP (76). Animal model studies also confirm that specific deletion of key signaling molecules like AMPKα1 in Tregs can lead to elevated autoantibody levels and aggravated tissue damage (77); Foxp3 gene-mutated scurfy mice directly demonstrate that complete deficiency of functional Tregs due to Foxp3 gene mutations spontaneously develop anti-BP180/BP230 autoantibodies, and some individuals even exhibit cutaneous vesicles (32, 70). Once Treg-mediated immune tolerance is broken, the previously suppressed autoreactive T cells are aberrantly activated. Among them, the loss of control over Th2 cells not only assists B cells in producing IgG4 and IgE subtype autoantibodies but also directly promotes the recruitment and activation of eosinophils by secreting cytokines such as IL-5 and IL-13 (73, 78, 79) Concurrently, the loss of control over Tfh cells is a key step driving autoantibody production: Treg functional defects can directly lead to a failure to control Tfh cell activity, exacerbating germinal center reactions (75, 80), which in turn leads to the massive production of pathogenic IgG antibodies (primarily IgG1 and IgG4 subtypes) targeting BP180 (mainly its NC16A domain) and BP230 (78). Specific peptides derived from BP180-NC16A (such as P2 and P5) can dominantly activate Th2 cells, induce IL-4 production, and drive B cells to secrete autoantibodies, directly proving the critical role of activated Th2 and Tfh cells in the autoimmune response of BP (81–83).

Direct immunofluorescence detection shows that pathogenic autoantibodies deposit linearly at the epidermal-dermal junction, simultaneously activating the complement system leading to C3 deposition. Deposited IgG1 subtype autoantibodies primarily activate complement via the classical pathway, while proteases released by eosinophils can directly cleave C3, amplifying the inflammatory response via the alternative pathway (84). The deposited immune complexes further activate the complement cascade, recruiting neutrophils, eosinophils, and mast cells to the site (85–87). Under normal conditions, Tregs can suppress the activation and protease release of these inflammatory cells by secreting inhibitory cytokines like IL-10 and TGF-β. They also reduce IL-17-driven neutrophil recruitment by inhibiting Th17 cells (73, 78). Th17 cells and their effector molecule IL-17 play an important role in BP pathogenesis: elevated levels of IL-17 are detected in BP patients, and IL-17 can significantly upregulate the expression of Matrix Metallopeptidase 9 (MMP-9) and neutrophil elastase, directly degrading BP180 and disrupting the basement membrane structure (88). Consequently, in the context of Treg functional deficiency, this suppressive effect is weakened, leading to overactivation of inflammatory cells and massive release of proteases such as MMP-9, neutrophil elastase, and tryptase. These proteases can directly degrade the extracellular domains of BP180 (e.g., MMP-9 and neutrophil elastase cleaving the NC16A region) and other basement membrane components (e.g., laminin-332, type IV collagen), while tryptase can indirectly exacerbate damage by activating proenzymes, among other mechanisms. Ultimately, the structural integrity of the BMZ is compromised, leading to separation of the epidermis from the dermis and the formation of the characteristic subepidermal blisters of BP. Given the central role of Treg functional defects in BP pathogenesis, restoring or enhancing their function has become a potential therapeutic strategy. Low-dose IL-2 therapy, by selectively expanding Tregs, has been shown to suppress autoantibody production and ameliorate disease symptoms (62, 89). Furthermore, genetically modified Treg therapies (such as CAR-Treg) also show broad prospects for precisely regulating B cell responses and inhibiting pathogenic antibody generation (90).

### 5.2 Recruitment of inflammatory effector cells and the regulatory role of Treg

In BP, the extensive infiltration of neutrophils (85), eosinophils (85), and mast cells (87) is a critical factor contributing to pathological damage. Tregs play a crucial role in modulating the inflammatory response by suppressing the overactivation of these effector cells. A diminished recruitment of Tregs to inflammatory sites directly undermines local immunosuppression, thereby exacerbating skin inflammation. In the BP-like epidermolysis bullosa acquisita (EBA) model (91), the administration of the retinoic acid receptor agonist Tamibarotene significantly reduced Treg recruitment to the dermis while prolonging neutrophil responsiveness to immune complexes, consequently worsening tissue damage. Similarly, 12/15-lipoxygenase-deficient mice exhibited prolonged and aggravated IgG-mediated BP-like inflammation due to inadequate Treg recruitment (72), which diminished the suppression of neutrophils. However, the supplementation of functional Treg cells reversed this process and significantly alleviated inflammation. Tregs primarily suppress the chemotaxis and activity of neutrophils and eosinophils by secreting anti-inflammatory cytokines such as IL-10 and TGF-β. Furthermore, Tregs can inhibit the formation of neutrophil extracellular traps (NETs) through the secretion of IL-10. NETs have the capacity to capture and activate autoantibodies and complement, thereby further amplifying local inflammation (92). A functional impairment of Tregs results in the failure of this inhibitory mechanism. When neutrophils are recruited to the BMZ and become activated, they release destructive enzymes, including neutrophil elastase and matrix metalloproteinase-9 (MMP-9). These enzymes can directly degrade structural proteins, such as BP180, leading to epidermal-dermal separation. Notably, the activity of neutrophil elastase is regulated by α1-antiprotease, which can be cleaved and inactivated during inflammatory processes (92). Therefore, the functional integrity of Tregs directly influences the extent of neutrophil-mediated terminal tissue damage.

### 5.3 Dysregulation of pathogenic T cell subsets by Treg cells

In the peripheral circulation and localized skin lesions of BP patients, an abnormal expansion of CD4+ T cell subsets, primarily consisting of Th2, Th17, and Tfh cells, has been observed. These cells, along with functionally impaired Tregs, form an effector network that drives disease progression (93). Th2 cells are the principal drivers of humoral immune responses, promoting B cell proliferation, inducing antibody class switching to IgG4 and IgE, and activating and recruiting eosinophils through the secretion of cytokines such as IL-4 and IL-5, thereby exacerbating inflammation (93). Research has confirmed that T cells in BP patients exhibit specific responses to the core antigen BP180-NC16A. These T cells display a memory phenotype and can simultaneously produce both Th1 and Th2 cytokines (94, 95). Notably, specific peptides derived from BP180-NC16A (e.g., P2 and P5) can dominantly activate Th2 cells, inducing IL-4 production and driving B cells to secrete autoantibodies, thereby underscoring the critical role of activated Th2 cells in the autoimmune response associated with BP (81). Under normal conditions, Treg cells effectively suppress the activation of Th2 cells and their cytokine production through the secretion of IL-10 and TGF-β. Consequently, the functional impairment of Treg cells leads directly to uncontrolled Th2 responses, exacerbating autoantibody production and eosinophilic inflammation. Th1 cells are also implicated in some BP patients, where the IFN-γ they secrete can activate macrophages and enhance antigen presentation, thus amplifying the immune response, although they typically do not play a dominant role (93). Treg cells play a crucial role in suppressing the activation of autoreactive T cells, including Th1 cells, through the secretion of inhibitory cytokines such as IL-10 and by competitively inhibiting the co-stimulatory signals from APCs via high expression of CTLA-4. This mechanism reduces the production of pro-inflammatory factors, including IFN-γ. During the active phase of BP, a decrease in the number or functional impairment of Treg cells results in diminished suppressive capacity, which may indirectly exacerbate Th1-related inflammation (84). The Th17-related cytokine IL-17 serves as a key effector molecule in inflammatory responses, with elevated levels detected in BP. Notably, the rapid and significant application of potent topical corticosteroids can concurrently reduce IL-17 expression and alleviate clinical symptoms of BP (96), suggesting a correlation between IL-17 levels and clinical prognosis. IL-17 is known to significantly upregulate the expression of matrix metalloproteinase 9 (MMP-9) and neutrophil elastase, which directly degrade BP180 and disrupt the basement membrane structure (88). *In vitro* studies have confirmed that the inhibition of IL-17A effectively blocks antibody-mediated epidermal-dermal separation. Under normal circumstances, Treg cells can effectively suppress the differentiation and function of Th17 cells by secreting cytokines such as IL-10 and TGF-β, thereby maintaining the balance between Th17 and Treg cells. The disruption of this balance is recognized as a common hallmark of inflammatory exacerbation in various autoimmune diseases (97–102). Therefore, the functional impairment of Tregs in BP is a key factor contributing to the excessive activation of the Th17 pathway, which subsequently drives neutrophilic tissue damage.

Tfh cells are a specialized subset of T cells that assist B cells in the production of high-affinity antibodies within the germinal center (GC). They promote B cell activation, proliferation, antibody class switching, and differentiation into plasma cells by expressing various molecules (including BCL6, CXCR5, PD-1, and ICOS) and secreting interleukin-21 (IL-21) (103, 104). Follicular tregs, as a special subset of tregs, co-express FOXP3 and CXCR5 and also localize to the germinal center. They specifically inhibit Tfh cell function through mechanisms such as CTLA-4-mediated contact inhibition and secretion of IL-10/TGF-β, negatively regulating humoral immune responses to maintain immune tolerance (83, 105, 106). The balance between Tfr and Tfh cells is crucial for preventing excessive production of autoantibodies. In BP patients, the frequency of Tfh cells (represented by CD4+CXCR5+PD-1+ and CD4+CXCR5+ICOS+cells) in peripheral blood and serum IL-21 levels are significantly elevated, both positively correlating with anti-BP180-NC16A antibody titers, and both significantly decrease after effective treatment. Using an anti-CD3 antibody-activated CD4+ T cell and B cell co-culture system, flow cytometry was employed to sort Tfh cells. Blocking IL-21 in this system inhibited BP patient B cells from secreting anti-BP180-NC16A antibodies, demonstrating that the Tfh-IL-21 axis is necessary for driving pathogenic autoantibody production (82). Corine Pérals et al. (107) (105) found that at baseline, BP patients had higher frequencies of circulating Tfh cells than healthy donors; these cells exhibited an activated phenotype with significantly elevated surface expression of HLA-DR, ICOS, and PD-1. Further subdividing circulating Tfh cells by flow sorting into Tfh1 (CXCR3+CCR6^−^), Tfh2 (CXCR3^−^CCR6^−^), and Tfh17 (CXCR3^−^CCR6+) subsets revealed an increased proportion of Tfh17 and a decreased proportion of Tfh1 in BP patients. The ratio of (Tfh2 + Tfh17) to Tfh1 positively correlated with disease severity (BPDAI score) and normalized after treatment. Scurfy mice with Foxp3 gene mutations completely lack functional Treg cells (including the Tfr subset), and their Tfh cells exhibit abnormal activation. Transferring purified scurfy Tfh cells into T cell-deficient nude mice can induce the recipient mice to produce autoantibodies against BP230 and detect linear C4 complement deposition at the skin basement membrane—typical pathological features of human BP; whereas transferring CD4+ T cells depleted of Tfh cells does not induce skin disease (75). Consequently, functional defects in Treg cells, particularly Tfr cells, result in insufficient suppression of Tfh cells and germinal center reactions, promoting excessive autoantibody production.

In summary, The imbalance of the Treg/Tfr-Tfh axis exacerbates the immune dysregulation in the germinal center, directly promoting the expansion of plasmablasts and plasma cells and the production of pathogenic antibodies against BP180 and BP230. The functional impairment of Tfr cells prevents them from effectively restraining the overactivation of Tfh cells. In the hyperactive germinal center response, activated Tfh cells, by secreting IL-21 and expressing co-stimulatory molecules such as ICOS, specifically induce B-cell proliferation and differentiation into plasmablasts and mature plasma cells. During this process, B-cell clones targeting BP180 (NC16A domain) and BP230 are specifically activated and undergo extensive expansion, ultimately differentiating into plasma cells capable of persistently secreting high-affinity anti-BP180/BP230 IgG (predominantly IgG1 and IgG4 subtypes). These pathogenic plasma cells can migrate to the peripheral circulation and skin lesion sites. They continuously secrete autoantibodies that deposit at the epidermal-dermal junction, becoming the core effector molecules that trigger subsequent complement activation and inflammatory cell recruitment. This establishes a key pathological bridge linking T-cell regulatory dysfunction to humoral immune abnormalities.

## 6 Factors modulating Treg function in BP

### 6.1 Age and genetic predisposition

Age is a fundamental factor influencing Treg function, which correlates with the epidemiological observation that BP predominantly affects elderly populations. Research indicates that aging is associated with a progressive decline in both the quantity and functionality of Treg cells. In aged mouse models, the suppressive capacity of Tregs is significantly reduced, with noted abnormalities in their phenotype, metabolic activity, and inflammatory control (108, 109). Mechanistically, aging may elevate oxidative stress in Tregs through the dysregulation of the DCAF1/GSTP1/ROS axis, which promotes their senescence and impairs their immunosuppressive function (109). While some studies suggest that age-related metabolic reprogramming may enhance Treg function in specific microenvironments (e.g., tumors), these effects are context-dependent (110). In the realm of autoimmunity, aging generally results in decreased regulatory efficacy of Tregs and impaired communication with effector cells, thereby disrupting immune tolerance (111, 112). This perspective on immunosenescence is crucial for understanding the high incidence of BP in the elderly population. Additionally, genetic background influences Treg development and homeostasis. Mouse models with varying genetic backgrounds exhibit significant phenotypic and functional differences in Tregs (113).

In humans, specific genetic variants can influence immune tolerance by affecting the development, stability, or surface molecule expression of Tregs. For instance, deficiencies in the FGL2 gene are linked to Treg dysfunction (114). Additionally, mutations in the FOXP3 gene directly cause IPEX syndrome, which is characterized by Treg deficiency or dysfunction (115). Genome-wide association studies further indicate that Treg-related genetic variations are associated with an increased risk of multiple autoimmune diseases (116). In the context of BP pathogenesis, genetic susceptibility may collectively undermine tolerance to cutaneous autoantigens by impairing Treg migratory capacity, suppressive function, or their interactions with effector cells, thereby heightening disease risk (108). In summary, the age-related decline in Treg functionality, combined with genetically determined Treg vulnerabilities, may have additive or synergistic effects that together form a critical intrinsic basis for the development of BP.

### 6.2 Drug-induced Treg dysfunction

Dipeptidyl peptidase-4 (DPP-4) inhibitors are widely utilized clinical hypoglycemic agents, and their administration is linked to the onset of DPP-4i-BP. Research indicates that DPP-4 inhibitors may modify the local immune microenvironment, influence the quantity and functionality of Treg cells, and disrupt immune homeostasis. In comparison to cBP, patients with DPP-4i-BP exhibit distinct alterations in Treg subsets, such as abnormal proportions of naive Tregs, and experience a shorter latency period. This suggests that drug-induced modulation of Tregs plays a pivotal role in the rapid disruption of tolerance (65, 117). Immune checkpoint inhibitors (ICIs) represent another class of drugs that significantly impact Treg cells. ICIs, including anti-PD-1/PD-L1 and anti-CTLA-4 antibodies, enhance anti-tumor immunity by blocking inhibitory pathways, but concurrently undermine Treg-mediated autoimmune tolerance. Studies have shown that ICI treatment can lead to a decrease in Treg numbers and functional suppression, accompanied by an increase in pro-inflammatory cytokines and heightened infiltration of inflammatory cells, thereby triggering various immune-related adverse events, including BP (117–119). The underlying mechanism involves the disruption of the cutaneous local tolerance network, which consists of Langerhans cells, tissue-resident memory T cells, and Tregs (76). Clinical observations suggest that for ICI-related adverse events, the administration of low-dose IL-2 to expand and restore Treg function exhibits therapeutic potential (67). These findings underscore that drug-induced interference with Treg function is a critical mechanism in the pathogenesis of drug-induced BP and offer insights for managing such complications through Treg modulation.

### 6.3 Environmental and physical factors

Physical stressors, including trauma, ultraviolet radiation, and surgical interventions, are recognized as triggering factors for BP. These stimuli can directly compromise the skin barrier and modify the local immune microenvironment. For instance, ultraviolet radiation can initiate cutaneous inflammatory responses, potentially diminishing the function of Tregs and impairing their recruitment to sites of inflammation, thereby disrupting local immune tolerance (108). Research has also demonstrated that in tissue injury models, specific chemokine axes, such as CXCL12-CXCR4, are activated to recruit Tregs for tissue repair (120). The potential dysregulation of this mechanism in the context of BP-related physical stress requires further investigation. Additionally, physical stress may influence the functional adaptability and stability of Tregs by affecting key transcription factors like TFEB and IRF4, as well as their metabolic state (121, 122). Chronic inflammation and comorbid conditions create a systemic environment that continuously affects Tregs. For example, metabolic disorders such as diabetes and obesity are frequently associated with chronic low-grade inflammation and lipid metabolism disorders, which can impair Treg function (123, 124). Moreover, gut microbiota dysbiosis, as a significant environmental factor, can influence the development and functionality of Tregs, contributing to systemic immune imbalances and being linked to autoimmune diseases, including BP (125). In a chronic inflammatory environment, persistently elevated levels of pro-inflammatory cytokines (e.g., IL-6, IL-17, TNF-α) can not only directly suppress Treg function but may also drive their conversion toward pro-inflammatory phenotypes, potentially leading to the loss of Foxp3 expression (126–129). Furthermore, comorbid conditions such as neurological disorders and malignant tumors indirectly affect Tregs through intricate immune networks, thereby disrupting systemic immune homeostasis (108, 130). Thus, external triggers, including environmental and physical factors, interfere with Tregs—an essential immunoregulatory hub—through multiple pathways. These factors synergize with intrinsic elements such as age, genetics, and medications, collectively contributing to the onset and progression of BP in susceptible individuals.

## 7 Conclusion and future perspectives

**Figure 1:**
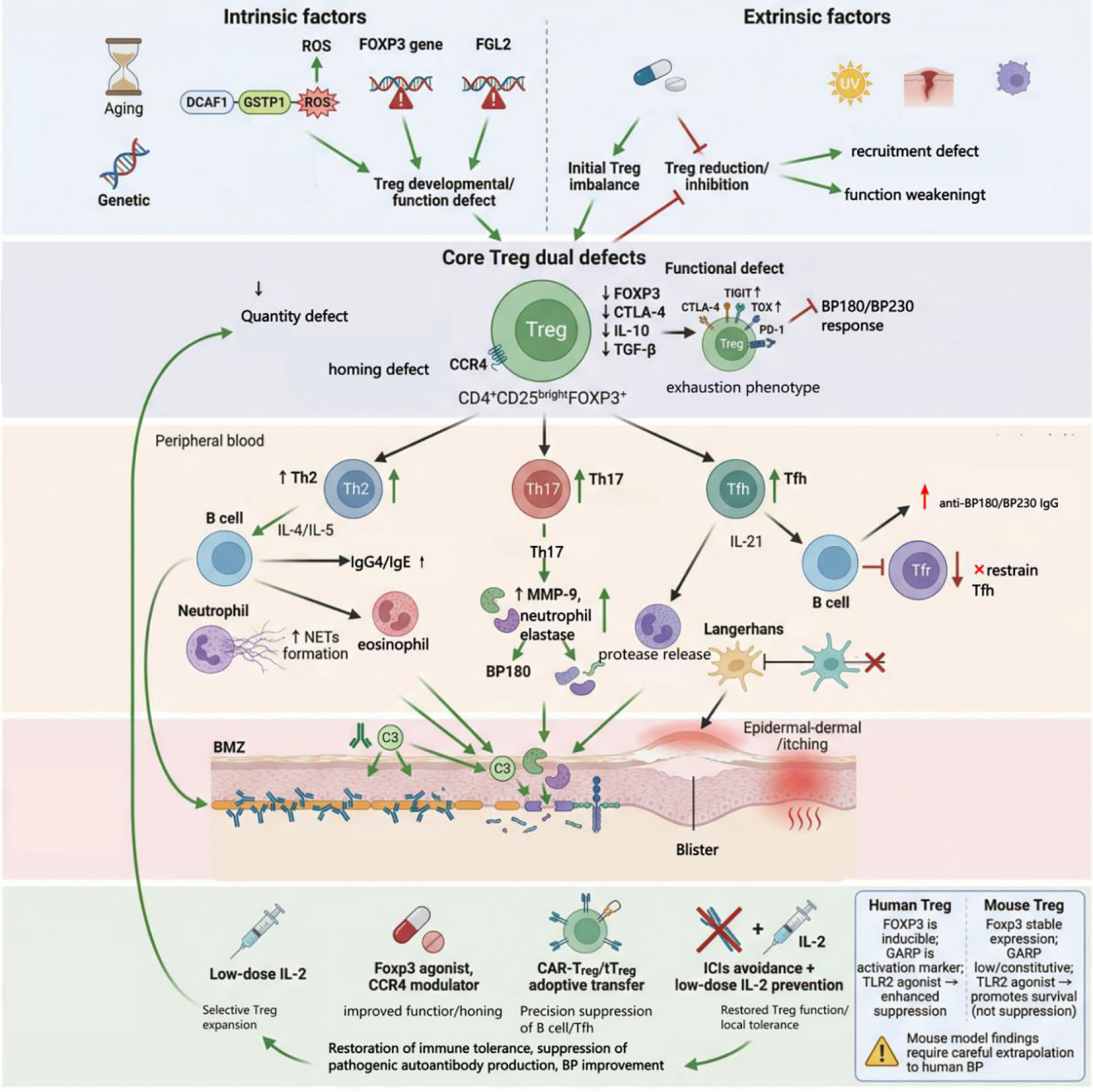
Key factors that impair Treg function and stability include intrinsic elements such as aging and genetic predisposition, as well as extrinsic factors, including specific medications, physical trauma, ultraviolet radiation, and metabolic disorders such as diabetes and obesity. Collectively, these factors weaken the immunosuppressive capacity of Tregs, leading to a reduction in the secretion of their critical inhibitory cytokines, TGF-β and IL-10. The loss of Treg function removes the suppression on various pathogenic helper T cell subsets, resulting in the aberrant activation of Th1, Th2, Th17, and Tfh cells, along with excessive secretion of their respective effector cytokines: IFN-γ, IL-4/IL-13, IL-17, and IL-21. This shift in the cytokine milieu, from anti-inflammatory to strongly pro-inflammatory, drives the production of BP autoantibodies, the infiltration of inflammatory cells, and ultimately leads to tissue damage.

BP is an autoimmune bullous disease with high incidence in the elderly. Its core pathogenesis involves immune tolerance imbalance at the dermal-epidermal junction, where Tregs play a key regulatory role. BP patients commonly exhibit abnormalities in both the number and function of Tregs. These abnormalities directly lead to the aberrant activation of pathogenic T cell subsets such as Th2, Th17, and Tfh cells, which promotes B cells to produce anti-BP180/BP230 autoantibodies. This activates the complement system and recruits inflammatory cells such as neutrophils and eosinophils, ultimately resulting in immune complex deposition at the BMZ and blister formation. Furthermore, age-related immunosenescence, genetic susceptibility, and exposure to drugs like DPP-4 inhibitors/immune-checkpoint inhibitors can impair Treg function. Environmental factors, such as ultraviolet radiation, trauma, and metabolic disorders, also contribute through pathways like oxidative stress and inflammatory microenvironment remodeling. Multiple factors synergistically contribute to the pathogenesis of BP (**Figure 1**). Restoring Treg quantity and function can effectively suppress the autoimmune response in BP, providing a core theoretical basis for targeted immunotherapy of BP.

Although significant progress has been made in research on the role of Tregs in BP, there are still many limitations in translating current findings into clinical practice, and numerous knowledge gaps remain to be filled in the field. First, species-specific differences pose a key obstacle to translation. Human and mouse Tregs exhibit significant differences in FOXP3 regulation, GARP expression, and TLR2 activation effects. Most existing BP models are mouse models involving passive antibody transfer or gene knockout, whose immune microenvironment and disease progression patterns differ significantly from human spontaneous BP. Some Treg regulatory mechanisms discovered in mice cannot be directly applied to humans. Second, clinical research findings are contradictory. Some studies observed an increase in Treg cells in the circulation or lesions of BP patients, contradicting the conclusion of Treg reduction in most studies. This is mainly due to issues such as inconsistent Treg detection markers, lack of stratification based on disease stage and treatment status of patients and disconnect between studies on peripheral blood and lesional skin. Additionally, key mechanisms in the field remain unclear. These include the upstream molecular regulatory pathways of Treg homing defects and functional exhaustion, the heterogeneity patterns of Treg abnormalities in different BP subtypes (traditional BP/drug-induced BP), the interaction mechanisms between Tregs and local skin immune cells such as Langerhans cells and neutrophils, and the regulatory role of Tfr cells on Tfh cells and germinal center reactions.

To address the aforementioned issues, future research needs to progress on multiple fronts to enhance the clinical translation value of findings. First, optimize experimental animal models by developing humanized immune mice incorporating human skin graft models to simulate the immune microenvironment and Treg phenotypic characteristics of human BP. Simultaneously, develop spontaneous BP models incorporating multiple factors such as age, genetics, and metabolic disorders to better align animal models with the pathogenic background of human disease. Second, standardize clinical research criteria. Clearly define CD4+CD25^bright^FOXP3+TNFR2+ cells as the core markers for detecting functional Tregs. Conduct stratified studies on BP patients based on different disease stages and treatment statuses and synchronously analyze the changing patterns of Tregs in peripheral blood and lesional skin to resolve contradictory research results. Third, utilize technologies like single-cell sequencing, spatial transcriptomics, and ChIP to deeply analyze molecular mechanisms. Screen key molecules and signaling pathways regulating Treg Foxp3 expression, phenotypic stability, and homing function, and clarify the interactions between Tregs and local skin immune cells. Finally, advance targeted therapy development. Optimize dosing regimens, treatment courses, and combination therapy strategies for low-dose IL-2 to expand Tregs and conduct clinical trials to verify their efficacy and safety. Simultaneously, based on molecular mechanism research, develop specific targeted drugs such as Foxp3 agonists and CCR4 modulators, and explore emerging therapeutic strategies like CAR-Treg adoptive transfer to provide precise immune regulation treatment options for BP. This study lays a theoretical foundation for Treg-targeted therapy in BP. Future breakthroughs in the aforementioned research directions are needed to promote the translation of basic research findings into clinical practice, providing new ideas for improving the prognosis of BP patients and reducing disease recurrence rates, and also offering important references for immune regulation therapy of other autoimmune skin diseases.
